# PITX2 gain-of-function induced defects in mouse forelimb development

**DOI:** 10.1186/1471-213X-8-25

**Published:** 2008-02-29

**Authors:** Johan Holmberg, Gorel Ingner, Curt Johansson, Peter Leander, Tord A Hjalt

**Affiliations:** 1 Department of Experimental Medical Science, Division for Cell and Matrix Biology, Lund University, Lund, Sweden; 2 Diagnostic Radiology, Malmö University Hospital, Lund University, Malmö, Sweden

## Abstract

**Background:**

Limb development and patterning originate from a complex interplay between the skeletal elements, tendons, and muscles of the limb. One of the genes involved in patterning of limb muscles is the homeobox transcription factor *Pitx2 *but its role in forelimb development is uncharacterized. Pitx2 is expressed in the majority of premature presumptive forelimb musculature at embryonic day 12.5 and then maintained throughout embryogenesis to adult skeletal muscle.

**Results:**

To further study the role of Pitx2 in forelimb development we have generated transgenic mice that exhibit a pulse of PITX2 over-expression at embryonic day 13.5 and 14.5 in the developing forelimb mesenchyme. These mice exhibit a distal misplacement of the biceps brachii insertion during embryogenesis, which twists the forelimb musculature resulting in severe skeletal malformations. The skeletal malformations have some similarities to the forearm deformities present in Leri-Weill dyschondrosteosis.

**Conclusion:**

Taken together, the tendon, muscle, and bone anomalies further support a role of Pitx2 in forelimb development and may also shed light on the interaction between the skeletal elements and muscles of the limb during embryogenesis.

## Background

Limb development and patterning originate from close interactions between tendon, cartilage and muscle precursor cells. Mouse forelimb development is first evident at about embryonic day (E) 9.5. Approximately 24 h later myogenic cells are identified at the base of the forelimb and at E11.5 the first hint of humerus is apparent [1]. At E14.5 a miniature model of the forelimb has been formed. Limb muscle precursors migrate from the lateral part of the somites into the limb bud where they undergo final differentiation. Among the transcription factors involved in early myogenesis are Pax3 and Lbx1 whose expression precedes the expression of myogenic regulatory factors (MRFs). MRFs belong to the MyoD family of basic helix-loop-helix factors. In mammals there are four such factors: Myf5, MyoD, myogenin, and MRF4 [2]. Little is known about the mechanisms whereby these genes regulate limb muscle development. The homeobox transcription factor *PITX2 *was originally identified as one of the genes responsible for Axenfeld-Rieger syndrome, mainly affecting eyes, teeth, and abdominal organs [3,4]. Pitx2 is expressed in a subset of Pax3^+ ^limb muscle precursors already at E10.5. By E12.5 Pitx2 is expressed in all limb musculature and persists until adulthood [5]. Still, Pitx2 null mutants form nearly all muscle anlagen even though several of these muscle anlagen are distorted, coupled with malformation of the part of the body to which they attach [6]. Muscles attach to bone through tendons. Limb tendon cells originate from lateral plate mesoderm and tendon progenitor cells are regionalized in the dorsal and ventral areas of the limb where they are mixed with muscle progenitor cells [7,8]. Compared to other mesodermal tissues, such as blood vessels, cartilage, bone, and muscles, very little is known about the early formation and role of tendons during development. Among the transcription factors identified in developing tendons are scleraxis, *Eya1*, *Eya2*, *Six1*, and *Six2 *of which *Eya1, Eya2*, *Six1 *and *Six2 *are also expressed in limb muscle precursors [7,9-11]. This parallel expression in myoblast precursors of somite origin and in mesenchymal cells derived from the lateral plate may ensure correct and concerted migration of the two cell types [12]. To further study the role of Pitx2 in forelimb development we have generated mice that exhibit a brief pulse of PITX2 over-expression in the forelimb mesenchyme from E13.5 to E14.5. The expression is driven by mouse keratocan (*Kera*) 5'-flanking sequence, which has been used previously to achieve over-expression of PITX2 in the cornea [13]. The construct was termed Ktcn-PITX2. Keratocan is one of three major components of the extracellular keratan sulfate proteoglycans present mainly in vertebrate corneal stroma but also expressed in non-ocular tissues such as skeletal muscle and tendon [14,15]. The *Kera *gene is expressed in limbs of mouse embryos at E13.5 and E14.5 [16,17]. This is the first report of PITX2 over-expression in the forelimb. The Ktcn-PITX2 mice exhibit PITX2 over-expression in the anterior forelimb mesenchyme extending from the humerus to the radius. The cells over-expressing PITX2 are of non-myogenic origin and co-express Six2. As Six2 is involved in tendon development, we hypothesize that the observed expression disturbs correct muscle insertion, which in the Ktcn-PITX2 mouse leads to a random left-right distal misplacement of the biceps brachii insertion. This in turn results in a 180 degrees twist of the forelimb musculature. The muscle and tendon anomalies also lead to severe skeletal malformations consisting of a shortened, thickened and malformed humerus, a bowed ulna and a deformed radius. These skeletal malformations have some similarities to the pathogenesis of Leri-Weill dyschondrosteosis, which is characterized by disproportionate short stature and a characteristic curving of the radius, known as the Madelung deformity [18]. In conclusion, these findings may increase our understanding about the role of Pitx2 in limb development and on the interactions between muscle, tendon, and bone during development.

## Results

### Ktcn-PITX2 mice have shortened, thickened and malformed humerus, deformed radius and bowed ulna

**Figure 1:**
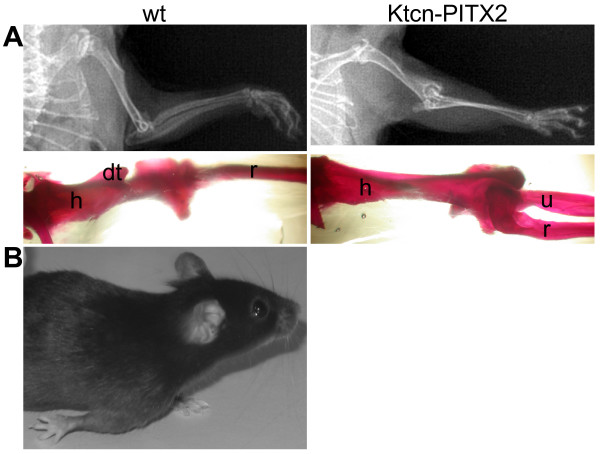
**Forelimbs of Ktcn-PITX2 mice exhibit severe bone malformations**. (A) X-ray images and Alizarin Red staining of wild type (left panel) and Ktcn-PITX2 (right panel) adult forelimbs. Right panel: Alizarin Red staining of Ktcn-PITX2 forelimb (ventral view) shows a diminished and misplaced deltoid tuberosity. The humerus and radius are malformed. Note the proximal bend of the radius, which results in a posterior misplacement. (B) A Ktcn-PITX2 mouse displays the characteristic forelimb phenotype. Abbreviations: dt, deltoid tuberosity; h, humerus; r, radius; u, ulna.

The Ktcn-PITX2 forelimb phenotype occurs randomly on either left or right forelimb. Occasionally both forelimbs are affected (Table 1). The bone malformations consist of a shortened, thickened and malformed humerus and a diminished deltoid tuberosity. The humerus malformation is most prominent distally and partly distorts the olecranon fossa. The olecranon fossa is a dorsal depression that receives the olecranon (elbow) of the ulna when the forearm is extended. This leads to a slight dislocation of the proximal ulna. Radius is bent proximally and approaches the elbow joint from a straight angel (Figure 1A). This locks the elbow in a simultaneous pronation-extension and forces the ulna to bend upwards to reach the carpals. The limitations in elbow movement in combination with the 180 degrees twist of the paw makes the forelimb insufficient to support on (Figure 1B). Hindlimbs, in contrast, do not exhibit any phenotype. The Ktcn-PITX2 mice are fertile and have normal life span.

**Table 1:** Distribution of phenotypes in 1-month-old wild type and Ktcn-PITX2 mice.

Normal	Mild (left/right/both)	Severe (left/right/both)	Total
*Wild type*			
15	0	0	15
*Ktcn-PITX2*			
10	30 (15/12/3)	36 (14/15/7)	76

### Limb malformations are present in E18.5 and in newborn Ktcn-PITX2 mice

**Figure 2:**
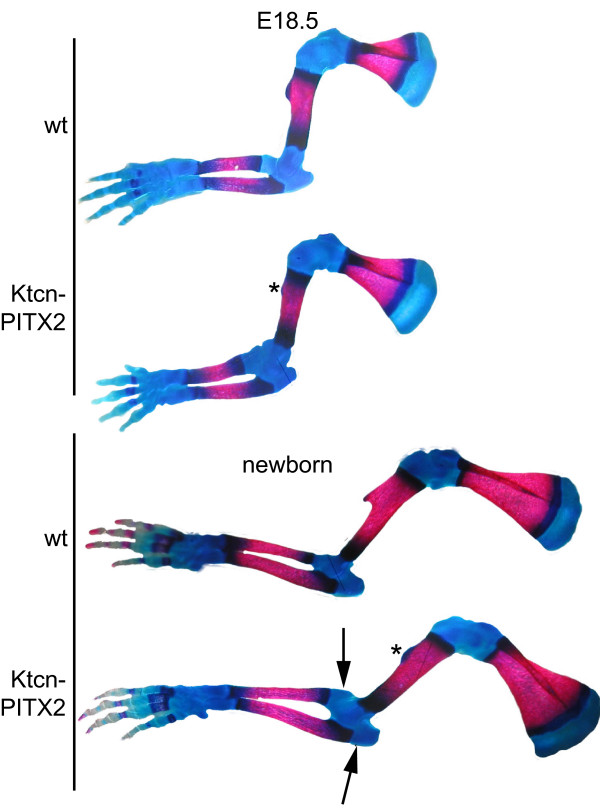
**Skeletal malformations are present in E18.5 and in newborn Ktcn-PITX2 mice**. Alizarin Red and Alcian Blue stainings of forelimbs from E18.5 (upper section) and newborn (lower section) mice. Forelimbs of E18.5 and newborn Ktcn-PITX2 mice display a diminished deltoid tuberosity (*). Newborn Ktcn-PITX2 mice also exhibit a thickening of the distal humerus, which tends to partly dislocate the radius anteriorly and also slightly dislocate the proximal part of the ulna in a more lateral-dorsal position, which makes the ulna appear twisted (arrows).

To investigate potential embryonic bone and cartilage anomalies we stained forelimbs from E18.5 and newborn mice with Alcian Blue and Alizarin Red. At E18.5 Ktcn-PITX2 mice display a diminished deltoid tuberosity and newborn Ktcn-PITX2 mice also exhibit a distal thickening of the humerus. The thickening tends to partly dislocate the radius anteriorly and also slightly dislocate the proximal part of the ulna in a more lateral-dorsal position, which makes the ulna appear twisted (Figure 2). Bone and cartilage zones were carefully examined at high resolution but appeared normal in the Ktcn-PITX2 forelimbs of all ages (data not shown).

### Ktcn-PITX2 mice over-express PITX2 in the forelimbs

**Figure 3:**
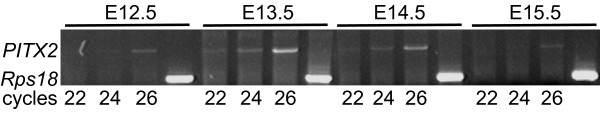
**Expression of human *PITX2 *mRNA in forelimbs of Ktcn-PITX2 mice**. Semi-quantitative RT-PCR of E12.5, E13.5, E14.5, and E15.5 Ktcn-PITX2 mouse forelimbs shows a *PITX2 *expression peak at E13.5-14.5.

**Figure 4:**
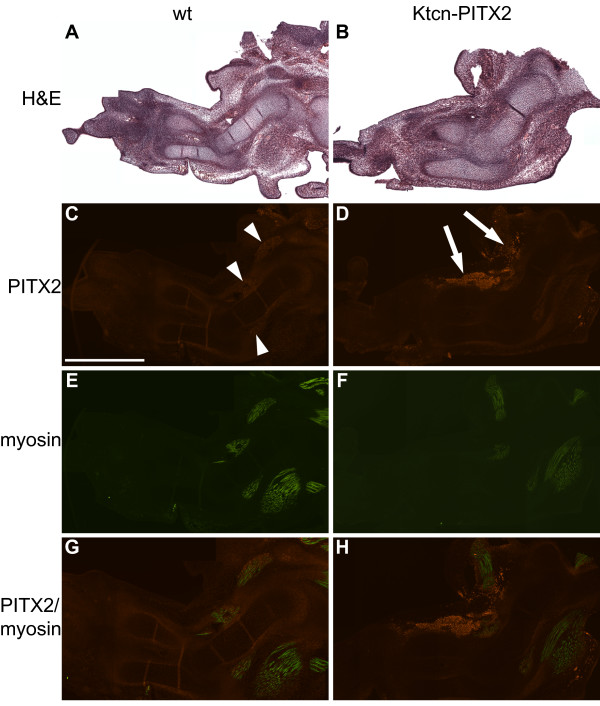
**PITX2 is over-expressed in non-myogenic cells**. (A and B) H&E stained sagittal paraffin sections of E13.5 wild type and Ktcn-PITX2 forelimbs. (C, E, G) Pitx2 is expressed in the musculature of wild type forelimbs (arrowheads) as evident from its co-expression with myosin. (D, F, H) PITX2 is over-expressed in the anterior mesenchyme reaching from the humerus to the distal part of radius (arrows). Note that the cells over-expressing PITX2 do not stain for muscle specific antibodies. Scale bar: 1 mm.

**Figure 5:**
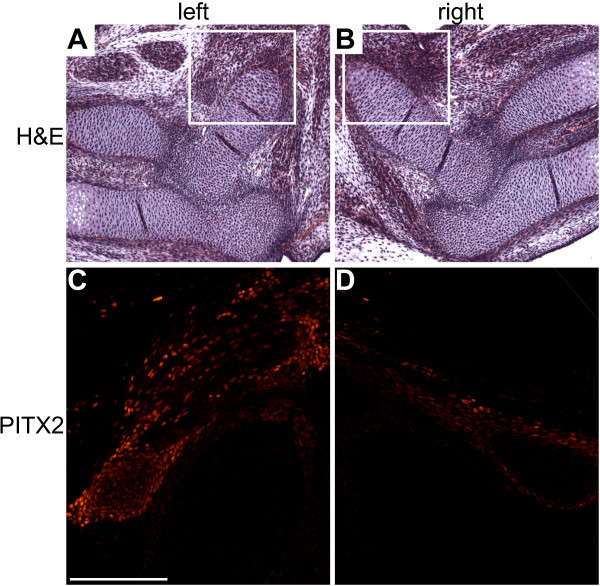
**PITX2 is over-expressed in both right and left forelimbs of the Ktcn-PITX2 mice**. (A and B) H&E stained sagittal paraffin sections of left and right forelimb from one individual E14.5 mouse. The marked areas are magnified in C and D. (C and D) Immunohistochemistry reveals PITX2 over-expression in both right and left forelimb. Scale bar: 0.5 mm.

**Figure 6:**
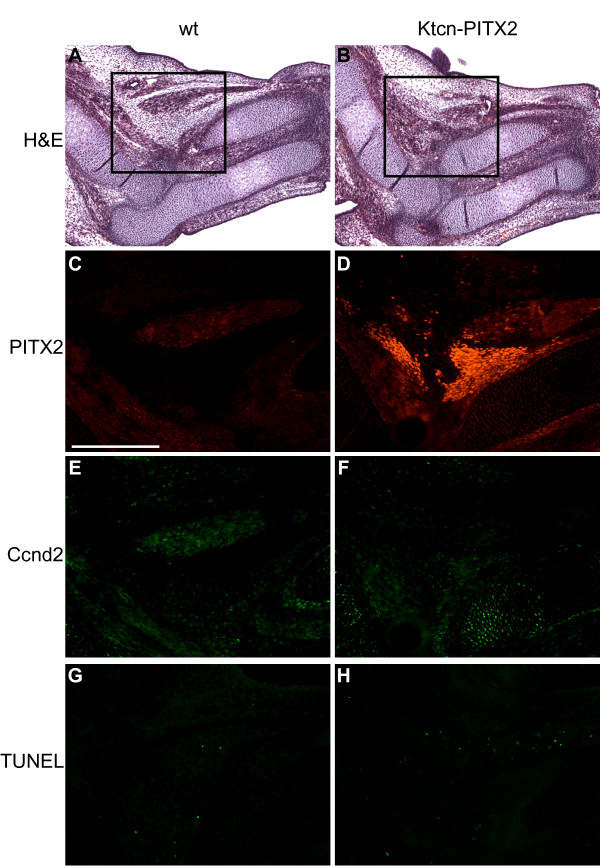
**Unchanged expression of Ccnd2 and no differences in apoptotic activity in the Ktcn-PITX2 forelimbs**. (A and B) H&E stained sagittal paraffin sections of forelimbs from E14.5 mice. The marked areas in A and B are magnified in C-H. (C-F) Double labeling immunohistochemistry reveals that the cells over-expressing PITX2 (D) do not display an increased expression of Ccnd2 (F). (G and H) TUNEL staining of sections from wild type and Ktcn-PITX2 forelimbs does not show any difference in apoptotic activity in the cells over-expressing PITX2. Scale bar: 0.5 mm.

The Ktcn-PITX2 mouse model was originally made to study the function of the homeobox gene *PITX2A *in eye development and its role in the pathology of Axenfeld-Rieger syndrome and glaucoma [13]. The Ktcn-PITX2 mice over-express human PITX2A in the cornea and iris under the *Kera *promoter and upstream regulatory elements, but *Kera *is also expressed throughout the limbs of embryos from E13.5 to E14.5 [17]. To study whether the Ktcn-PITX2 mice express human *PITX2 *mRNA in the forelimbs at the same embryonic stages as *Kera *is expressed we isolated forelimb RNA from Ktcn-PITX2 embryos (E12.5, E13.5, E14.5, and E15.5) and performed semi-quantitative RT-PCR. Human *PITX2A *mRNA expression level peaked at E13.5 and E14.5 and declined thereafter, consistent with the expression pattern of *Kera *(Figure 3). Further, we used immunohistochemistry to study the expression pattern of PITX2 protein in the forelimbs at E13.5. In wild type embryos Pitx2 expression is restricted to well defined anterior and posterior muscle groups consistent with previous observations (Figure 4C,E,G) [5]. In the Ktcn-PITX2 forelimbs we observed a proximal anterior over-expression of PITX2 in the mesenchyme close to the humerus but also an anterior over-expression in the mesenchyme expanding from the elbow joint to the distal part of radius. These areas did not stain for muscle specific antibodies against Pax3, MyoD1, myogenin, or myosin (Figure 4D,F,H and data not shown). This could either be due to that the cells over-expressing PITX2 are not of myogenic origin, or muscle markers are down regulated as a result of the over-expression. *In vitro *studies have shown that Pitx2C over-expression promotes cell proliferation and arrests differentiation in myoblasts [19]. However, in the forelimbs of adult Ktcn-PITX2 mice, all muscle groups are present, indicating that the cells over-expressing PITX2 are of non-myogenic origin. In the Ktcn-PITX2 mice, we consistently observed PITX2 over-expression in both right and left forelimbs (Figure 5C,D). It has been reported that Pitx2 regulates the expression of cyclin D2 (Ccnd2) after Wnt induction in C2C12 myoblasts [20]. We did not detect any increased expression of Ccnd2 in the cells over-expressing PITX2 or any increased apoptotic activity (Figure 6). Notably, the region of over-expression is situated close to where the first phenotypes later appear.

### The biceps brachii of Ktcn-PITX2 mice has a misplaced insertion

**Figure 7:**
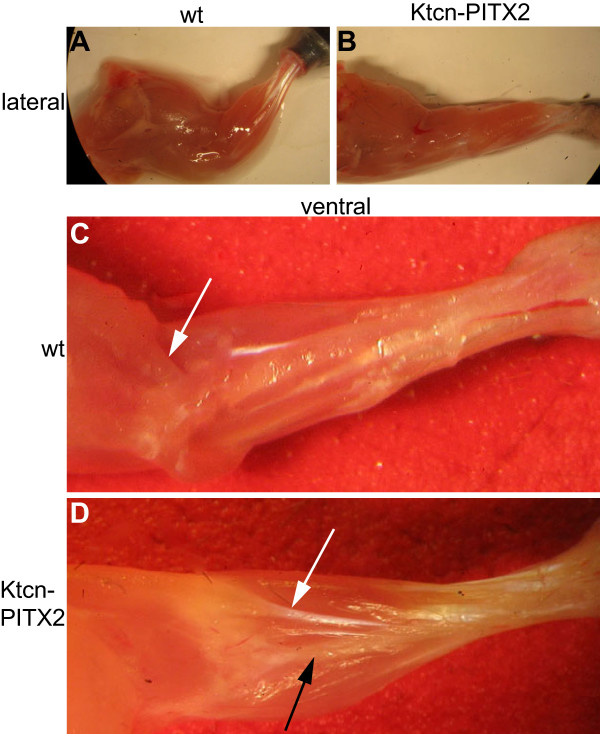
**Forelimbs of adult Ktcn-PITX2 mice display tendon anomalies**. (A and B) The adult Ktcn-PITX2 limb musculature is twisted 180 degrees compared to wild type. Still, all muscle groups are present. (C) Normally, biceps brachii inserts at the proximal part of the radius (white arrow). (D) In the Ktcn-PITX2 mice biceps brachii insertion is altered (white arrow) as its distal end is curled around the radius (black arrow) and inserts dorsally at the very most distal part of the bone.

To gain a deeper understanding of the forelimb phenotype we dissected forelimbs from adult Ktcn-PITX2 mice. Despite the obvious twist of the musculature, all muscle groups are present and originate and insert correctly except for biceps brachii. Biceps brachii is a flexor muscle whose function is to decrease the joint angle at the elbow but also to supinate (turn palm superiorly or anteriorly) the forearm at the radioulnar joints. It originates at two separate sites in the scapula and inserts at the proximal part of the radius. In the Ktcn-PITX2 mice biceps brachii insertion is altered as its distal tendon is curled around the radius and inserts dorsally at the very most distal part of the bone (Figure 7). This would explain the limited elbow motion but also the permanent pronation of the Ktcn-PITX2 forelimbs. Muscle groups work as antagonists. If the supination movement is impaired its antagonist, the pronator, will dominate, which may result in the pronation position.

### PITX2 and Six2 are co-expressed in non-myogenic cells in the Ktcn-PITX2 forelimbs

**Figure 8:**
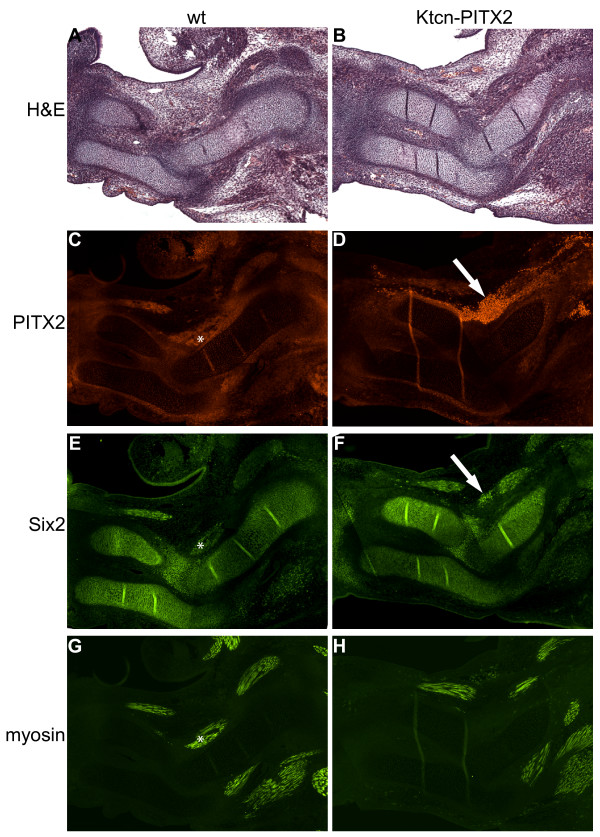
**At E13.5 PITX2 and Six2 are co-expressed in a limited numbers of non-myogenic cells**. (A and B) H&E stained sagittal sections of E13.5 wild type and Ktcn-PITX2 forelimbs. (C-H) At E13.5 both Pitx2 and Six2 are expressed in the developing forelimb musculature (*). (D, F) In the Ktcn-PITX2 forelimb, Six2 is also expressed in a limited numbers of the non-myogenic cells over-expressing PITX2 (arrow). Scale bar: 1 mm.

**Figure 9:**
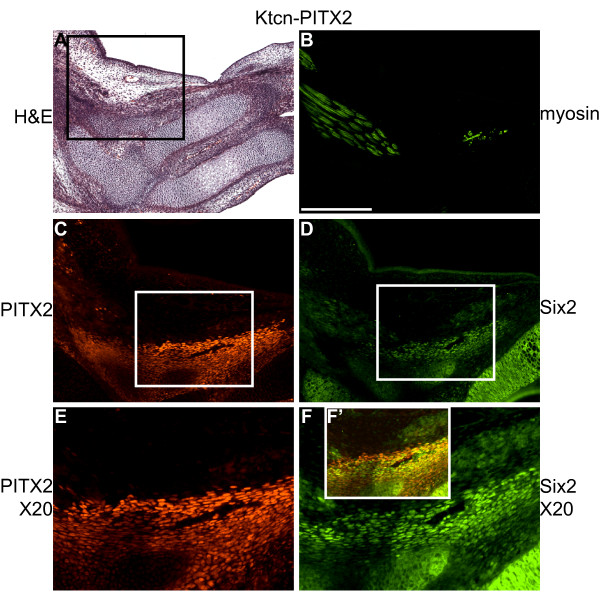
**At E14.5 PITX2 and Six2 are fully co-expressed in non-myogenic cells**. (A) H&E stained sagittal section of E14.5 Ktcn-PITX2 forelimb. The marked area is magnified in B-D. **(**B-D) At E14.5 Six2 expression overlaps a substantial area of the non-myogenic cells over-expressing PITX2. The marked areas are further magnified in E-F and overlaid in F'. Scale bar: 0.5 mm.

To study the molecular mechanism behind the Ktcn-PITX2 forelimb tendon anomalies and to further characterize the identity of the cells over-expressing PITX2, we analyzed the expression of Six2 in wild type and Ktcn-PITX2 forelimbs. At E13.5 both Pitx2 and Six2 are expressed in the developing forelimb musculature (Figure 8C,E), but in the Ktcn-PITX2 embryos, Six2 is also expressed in a limited area within the non-myogenic cells over-expressing PITX2 (Figure 8D,F). This co-expression is more evident 24 h later. At E14.5 Six2 expression overlaps a substantial area of the cells over-expressing PITX2 (Figure 9C–F). This suggests a relationship between Pitx2 and Six2 and indicates that at least part of the cells over-expressing PITX2 are involved in tendinous patterning. In addition, studies performed *in silico *show several potential Bicoid/Pitx2 binding sites in the *Six2 *promoter (data not shown).

## Discussion

Pitx2 acts in hindlimb development together with Pitx1 where the former specifies laterality and the latter determines hindlimb identity [21]. During forelimb development Pitx2 is expressed in almost all muscle anlagen from embryogenesis until adulthood [4,5,22-24]. We show here for the first time that over-expression of PITX2 in non-myogenic cells of the developing forelimb results in a distal misplacement of the biceps brachii insertion, which gives rise to severe bone malformations, randomly affecting left or right forelimb. The bone anomalies include a diminished deltoid tuberosity, a shortened, thickened and malformed humerus. In addition, it causes the radius to approach the elbow via a perpendicular bend, which in turn limits the elbow movement, and forces a dorsally dislocated ulna to curve over radius to reach the carpals. The phenotype worsens two to three weeks after birth, possibly due to the increased load on the forelimbs as the mouse starts to walk. On the other hand we frequently detected mice with both forelimbs affected from early on. Also, mice with only one affected leg never developed any phenotype on the supporting leg. The skeletal malformations have some similarities to the pathogenesis of Leri-Weill dyschondrosteosis, characterized by disproportionate short stature, curving of the radius, and subluxation of the distal end of the ulna. Mutations in the SHOX gene have been shown to cause the disorder. However, for 27% of the cases, no causative gene has been identified [25]. Pitx2 is expressed in the left lateral plate mesoderm and acts as an effector for left-right asymmetry in mesoderm derived organs such as lungs [26]. Also, *Pitx1*^-/- ^embryos show left-right asymmetry in the severity of a hindlimb phenotype due to redundancy between Pitx1 and Pitx2 [21]. Still, the limbs are symmetrical. It has previously been shown that function of Pitx2 is dosage-sensitive [13,27,28]. Hence, the more plausible explanation for the random left-right forelimb anomalies of the Ktcn-PITX2 mice could be minor differences in PITX2 expression levels between left and right. Pitx2 expression in limbs is restricted to muscle lineages and it has recently been shown that Pitx2 is one of the most complete muscle markers [5]. Immunohistochemistry on wild type forelimb sections demonstrate co-expression of Pitx2 and other muscle markers such as MyoD, myogenin, and myosin. In contrast, the cells over-expressing PITX2 in the Ktcn-PITX2 forelimbs do not express any of these muscle markers. Based on the observed tendon anomalies in the Ktcn-PITX2 forelimbs we stained for Six2, normally expressed in developing tendons. Co-expression of PITX2 and Six2 could indicate that the cells over-expressing PITX2 are involved in tendon development and positioning. The factors regulating correct attachments of tendons to bone and the time of commitment of tendon precursors to a tendon cell fate in vertebrates are largely unknown [29]. However, reports have shown that myotubes can induce tendon primordial into individual tendons and, moreover, if tendon precursors are removed, myotubes attach ectopically [30]. It is possible that the observed expression of Six2 in the cells over-expressing PITX2 interferes with the signals coordinating correct tendon insertion. Based on the observed co-expression of PITX2 and Six2 in our mouse model it is intriguing to note that both transcription factors are expressed during other developmental processes. Neural crest cells that migrate to the periocular mesenchyme during eye development express both Pitx2 and Six2 [31,32]. In addition, Eya1, another limb tendon marker, is expressed in developing anterior chamber structures of the eye including the iris, ciliary structures, and cornea, tissues that also express Pitx2 [12]. It would be interesting to further study a possible relation between Pitx2, Six2, and Eya1. Notably, Pitx1 is closely related to Pitx2 and normally involved in and restricted to hindlimb development. When ectopically expressed in the forelimbs it induces transformation and translocation of specific muscles, tendons, and bones of the forelimb [33]. Finally, the temporal overlap of muscle and tendon formation with the over-expression of PITX2 makes the Ktcn-PITX2 mouse an appropriate animal model to study the development of the musculoskeletal system.

## Conclusion

Over-expression of PITX2 during mouse forelimb development results in severe tendon, muscle, and bone anomalies. These observations further support a role of Pitx2 in forelimb development. This animal model may also be valuable for future studies on the interaction between the skeletal elements and muscles of the limb during embryogenesis and it could provide information on new regulatory pathways involving PITX2.

## Methods

### Mice

Transgenic animals were previously described [13]. The local ethics committee for animal research has approved of our experiments (Lund district). The mice were housed and treated according to guidelines of national and local animal ethics guidelines at the BMC animal facilities at Lund University, Lund.

### RNA isolation and RT-PCR

We used Trizol (InVitrogen, Carlsbad, CA) and RNeasy MinElute Clean-up (Qiagen, Valencia, CA) kits to isolate total RNA from limbs. Tissues were whole forelimbs frozen in liquid nitrogen, grinded on dry ice, and further homogenized in Trizol with syringes. RNA was purified with phenol/chloroform, precipitated, and treated with DNA-*free *(Ambion, Austin, TX) to remove contaminating DNA. Reverse transcription was performed using SuperscriptII (Gibco/BRL/InVitrogen, Carlsbad, CA). We used a mixture of random hexamer and oligo-dT primers (Promega, Madison, WI) according to the recommendations of the manufacturer. We used an amplicon for ribosomal binding protein 18 (*Rps18*) as a loading control. *PITX2A *and *Rps18 *were amplified as described [13].

### Histological techniques

Limbs were fixed in 4% formaldehyde in PBS and then embedded in paraffin according to standard procedures. Sections of 5 μm were mounted on slides. Sections for immunohistochemistry were deparaffinized and boiled in 0.1 M sodium citrate and 0.05% Tween 20 for 15 minutes prior to over night primary antibody staining. Antibodies were against PITX2A [32], cyclin D2 (Abcam, Cambridge, UK), myosin (My32), MyoD1 (SIGMA, Saint Louis, MO), Pax3, myogenin (F5D) (Developmental Studies Hybridoma Bank, Iowa City, IA), and Six2 (Affinity Bioreagents, Golden, CO). We used goat anti-rabbit Alexafluor 546, goat anti-mouse 488, and rabbit-anti-goat Alexafluor 546 as secondary antibodies (Molecular Probes, Eugene, OR). Slides were mounted in Permafluor (Beckman-Coulter, Fullerton, CA).

### Terminal dUTP nick-end labeling (TUNEL) assay

We used the *In situ *cell death detection kit fluorescein (Roche, Indianapolis, IN), to test apoptotic activity on 5 μm paraffin sections according to the recommendations of the manufacturer. Slides were mounted in Permafluor (Beckman-Coulter) and viewed in a fluorescence microscope.

### Bone and cartilage staining

Embryos and newborn animals were skinned, eviscerated and fixed in 95% ethanol for one week. The specimens were then placed in acetone for one week, stained in 1 volume 0,1% filtered alizarin red S (SIGMA), 1 volume 0,3% filtered alcian blue 8GX (SIGMA), 1 volume 99.8% acetic acid, and 17 volumes 70% ethanol for one week, cleared in 1% KOH, destained in 20% glycerol + 1% KOH, and transferred through a graded glycerol series and stored in 100% glycerol.

### X-ray imaging

The mice were sacrificed before X-ray exposure. A clinical Arco Ceil, Arcoma, Generator CPI Indico 100, and Canon CXDI-31 Portable DR System were used. Exposure settings were: 46 kV, 1.6 mAs.

## Authors' contributions

JH helped with experimental design, performed the experiments, and drafted the manuscript. GI, CJ, and PL carried out the X-ray imaging and helped to draft the manuscript. TAH conceived and designed the experiments and helped to draft the manuscript. All authors read and approved the final manuscript.
